# Plate-related results of opening wedge high tibial osteotomy with a carbon fiber reinforced poly-ether-ether-ketone (CF-PEEK) plate fixation: a retrospective case series of 346 knees

**DOI:** 10.1186/s13018-019-1514-1

**Published:** 2019-12-27

**Authors:** Claudia Hartz, Ralph Wischatta, Eckhardt Klostermeier, Malte Paetzold, Klaus Gerlach, Frank Pries

**Affiliations:** Department Arthroskopische Chirurgie und Sporttraumatologie, Mare Med , Eckernfoerder Strasse 219, 24119 Kronshagen, Germany

**Keywords:** Open wedge high tibial osteotomy (owHTO), PEEKPower HTO plate, Hinge fractures

## Abstract

**Background:**

While open wedge high tibial osteotomy (owHTO) is an established standard procedure to treat medial osteoarthritis of the knee in combination with varus deformity, it bears the risk of postoperative hardware failures and lateral cortical hinge fractures. This in turn can lead to an accelerated osteoarthritis, non-union, or a loss of correction accuracy. The purpose of the study was to evaluate the radiologic outcomes of owHTO with a carbon fiber reinforced poly-ether-ether-ketone (CF-PEEK) plate fixation in patients with medial osteoarthritis and varus deformity.

**Methods:**

Three hundred twenty-four consecutive patients (346 knees) who were treated with owHTO using the PEEKPower HTO plate were included in this retrospective study; 89.9% of the patients were overweight or obese. Patients were followed by conventional radiographs over a 12-month period. Typical plate-related results such as the time and quality of gap healing as well as the correction accuracy were analyzed. Furthermore, the number of lateral cortex fractures was determined.

**Results:**

Bony consolidation was observed after a mean gap healing time of 4.0 ± 1.7 months independent on the patients’ weight (*p* = 0.2302). With increasing gap sizes, bony healing was significantly prolonged (*p* < 0.001). Additionally, patients with greater gap sizes had a significantly increased risk for a lateral cortex fracture (*p* = 0.0041). However, none of the patients had a non-union 1 year postoperative. A hinge fracture occurred in 30% of patients. Hinge fractures with Takeuchi grades I and II increased the gap healing time compared to no fracture (*p* = 0.0069 and *p* = 0.0002, respectively), but only 1.2% of patients with hinge fracture had a clinical relevant loss of correction ≥ 3 mm. No implant failures were found.

**Conclusions:**

Open wedge HTO using the PEEKPower HTO plate for patients with medial osteoarthritis of the knee in combination with tibial varus deformity leads to excellent bony consolidation also in cases with a hinge fracture, a gap size > 12 mm as well as for severely obese patients.

## Background

Open wedge high tibial osteotomy (owHTO) is an established surgical procedure to treat patients with medial osteoarthritis of the knee in combination with varus deformity [1–3]. By reducing the effective load through the medial compartment and eduction or reversion of the adductor moment, the procedure aims to decelerate the degeneration progress, to relieve pain, and to correct the lower extremity alignment. While owHTO is a standard procedure, it bears the risk of postoperative hardware failures and lateral cortical hinge fractures [4–6]. Different clinical studies reported lateral hinge fractures in 18–39% of patients [7–10], which in turn can lead to an accelerated osteoarthritis, non-union, or a loss of correction accuracy [6, 11, 12]. Requirements for fixation systems in owHTO are high. They have to withstand the high forces of early full weight-bearing programs and minimize the risk of delayed and non-union in case of a lateral cortex fracture. At the same time, they have to allow for sufficient interfragmentary movement (IFM) to achieve a faster gap healing time [11, 13, 14]. In the past, several angle-stable locking plates, such as the Position HTO plate (Aesculap, Tuttlingen, Germany), the Puddu Plate (Arthrex Inc., Naples, FL, USA), or the TomoFix Plate (Synthes Medical, Oberdorf, Switzerland), have been developed aiming for an increased loading capacity and residual stability after failure of the lateral cortex [13]. Clinical results for patients treated with these implants have demonstrated a high stability and lower rates for loss of correction [5, 15, 16]. However, other studies have shown that a high stiffness of a locking plate may suppress callus formation and fracture healing [17, 18]. The authors suggested increasing the IFM of the implants in order to avoid incomplete bone healing.

Addressing the disadvantages of present implants for owHTO the PEEKPower HTO plate (Arthrex Inc., Naples, FL, USA) was introduced. The plate consists of carbon poly-ether-ether-ketone reinforced fibers. A biomechanical study, comparing the Tomofix plate composed of titanium to the PEEKPower HTO plate, showed that the PEEKPower HTO plate resists higher dynamic loadings and had a higher static flexural rigidity [19]. However, when followed-up in a clinical setting the 1st generation PEEKPower HTO plate resulted in a higher rate of implant-related complications than the TomoFix plate after 24 months [20]. This finding led to the development of the 2nd generation PEEKPower HTO plate. An initial prospective study provided promising outcomes of the 2nd generation PEEKPower HTO plate, showing comparable complication rates to commonly used fixation systems [21]. The study is, however, limited by the low number of patients (*n* = 28) and a maximum osteotomy gap size of 12 mm. Also, reliable mid- to long-term clinical outcomes of the 2nd generation PEEKPower HTO plate are missing.

The aim of this study was to evaluate the radiologic outcomes and safety of the 2nd generation PEEKPower HTO plate for valgus owHTO. Retrospectively, we analyzed the number of lateral cortex fractures, the quality and time of gap healing, the correction accuracy, and implant-related complications of 324 patients (346 knees) with a follow-up of 1 year after owHTO with the PEEKPower HTO plate.

## Methods

The ethics committee of the University of Lübeck approved this retrospective study. The postoperative rehabilitation program and the radiographic evaluation after owHTO were standard of care procedures for all patients. After implant removal, the medical treatment ended. For the analysis of the outcomes, all personal patient data were anonymized.

### Patients

Since 2010, the 2nd generation PEEKPower HTO plate (Arthrex Inc., Naples, FL, USA) is the standard plate for open wedge valgus HTO at our institution. No other plates are being used for this procedure.

Between 2010 and 2015, three surgeons performed 346 owHTOs in 324 consecutive patients. The vast majority of surgeries (79%) was done by the senior author (FP).

**Fig. 1:**
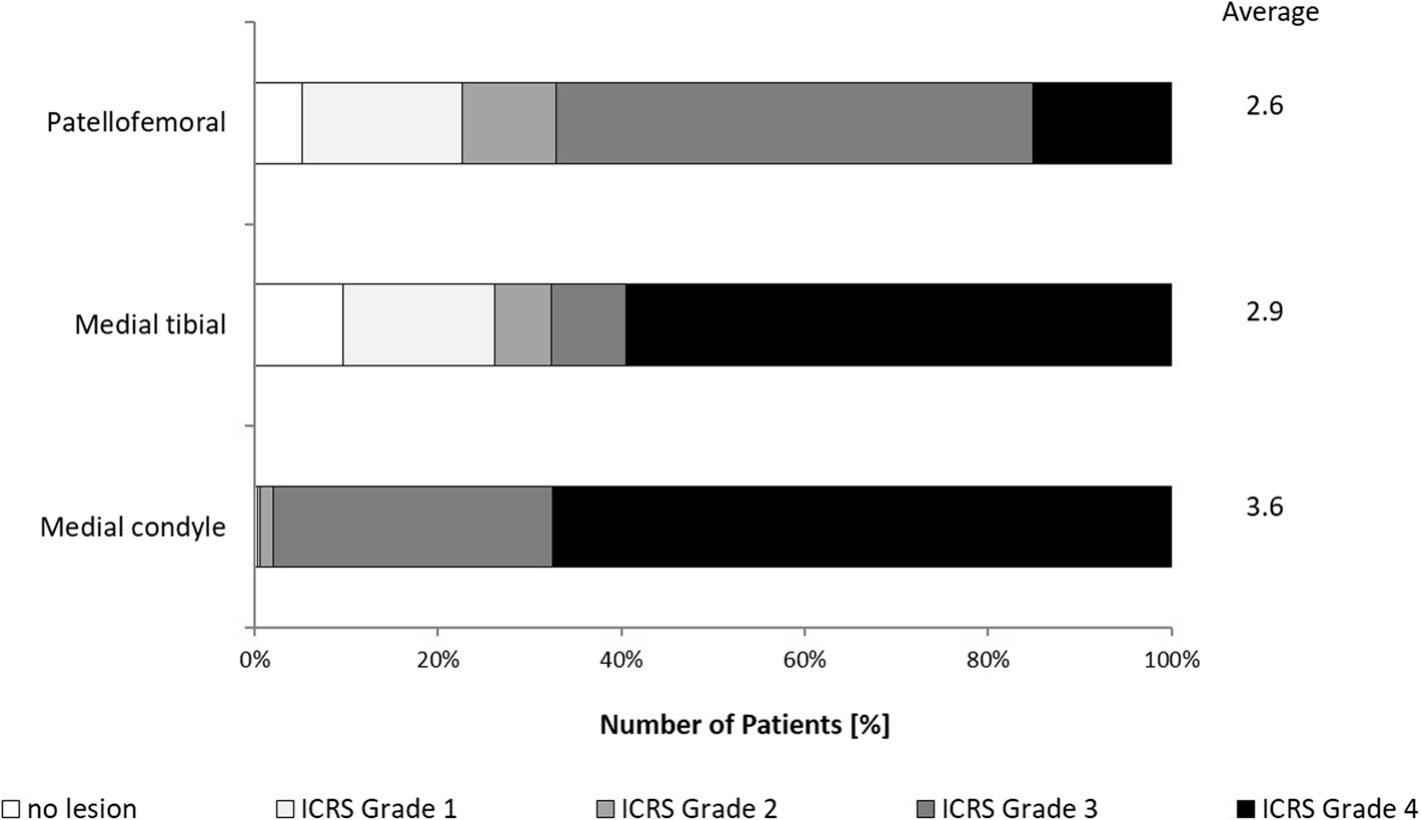
Cartilage lesions in the medial knee compartment of the study patients. The cartilage lesions were classified according to the ICRS (International Cartilage Repair Society) Hyaline Cartilage Lesion Classification System (grades 1–4)

Two hundred twenty-nine of the patients (69.4%) were males. The average age was 55 ± 8.9 years (range 30–76 years) and the mean weight 95 ± 16.5 kg (range 61–169 kg). Prior to owHTO, all patients underwent arthroscopic surgery. Cartilage lesions were classified according to the ICRS Hyaline Cartilage Lesion Classification System of the International Cartilage Repair Society (ICRS) (Fig. 1) from grades 0 to 4.

The inclusion criterion was symptomatic varus malalignment with a cartilage defect in the medial compartment of the knee. Exclusion criteria were pain in the lateral compartment with a cartilage defect > 1 according to the ICRS classification, the absence of the lateral meniscus, and high-grade medial and lateral ligamentous instability. None of the patients had additional ligamentous instability.

All cartilage lesions grades 3 and 4 seen in the medial and patellofemoral compartment were additionally treated with a bone marrow stimulation technique using microfracture or abrasion arthroplasty. Debridement was performed for cartilage lesion grade 2 to reduce mechanical stress.

### Surgical procedure

After arthroscopic knee surgery, owHTO using the 2nd generation PEEKPower HTO plate was performed as published [16, 22–24]. In 274 cases, the surgeon performed a uniplanar osteotomy and a biplanar osteotomy in 72 cases. The weight-bearing line was placed at the Fujisawa point (62% of the transverse diameter of the tibial joint line) and 1.5° valgus (recommendation of Noyes) according to the preoperative planning. An image converter was used during surgery to measure the Mikulicz line with the help of a taut cable. The gap was filled with ß-tricalcium phosphate (Osferion, Arthrex Inc., Naples, FL, USA) in 306 patients (85.5%). Time of plate removal was determined dependent on bony healing, swelling, return to sports, and activity of daily living. Twelve months after the osteotomy, the implant was removed in 97% of the patients.

### Rehabilitation

During the postoperative rehabilitation program, patients were treated with a straight immobilizer for 6 weeks. From day 1 to week 4 after surgery, passive-free motion during physiotherapy was allowed adapted to pain with a limited flexion of 90°. Weight-bearing of 20 kg was allowed from weeks 1 to 3. Afterwards, weight-bearing was increased to 40 kg, up to full weight-bearing after 6 weeks.

### Radiographic evaluation

For the preoperative planning of the osteotomy, the computer software CARESTREAM Vue PACS (Carestream Health Inc., NY, USA) was used. Standing long-leg as well as lateral and fixed-flexed posterior-anterior (PA) (Rosenberg view) radiography was performed. The osteotomy gap size was identified using the Miniaci preoperative planning technique during surgery [25].

#### Bone healing

Postoperative posterior-anterior and lateral radiographs were obtained at 1, 2, 3, and 12 months after owHTO to observe bone healing (standard of care). In case of non-completed bone healing after 3 months, radiography was performed additionally at 4, 5, 6, and 9 months after surgery, respectively. The assessment of radiological data was conducted by three independent observers. According to Apley, Solomon’s and Brosset criteria [26, 27], osseous bone healing was considered as being completed if the patient no longer had pain over the osteotomy gap at the time of full weight-bearing. Furthermore, the bridging callus had to reach 75% of the diameter of the osteotomy gap [26, 27].

#### Correction accuracy

**Fig. 2:**
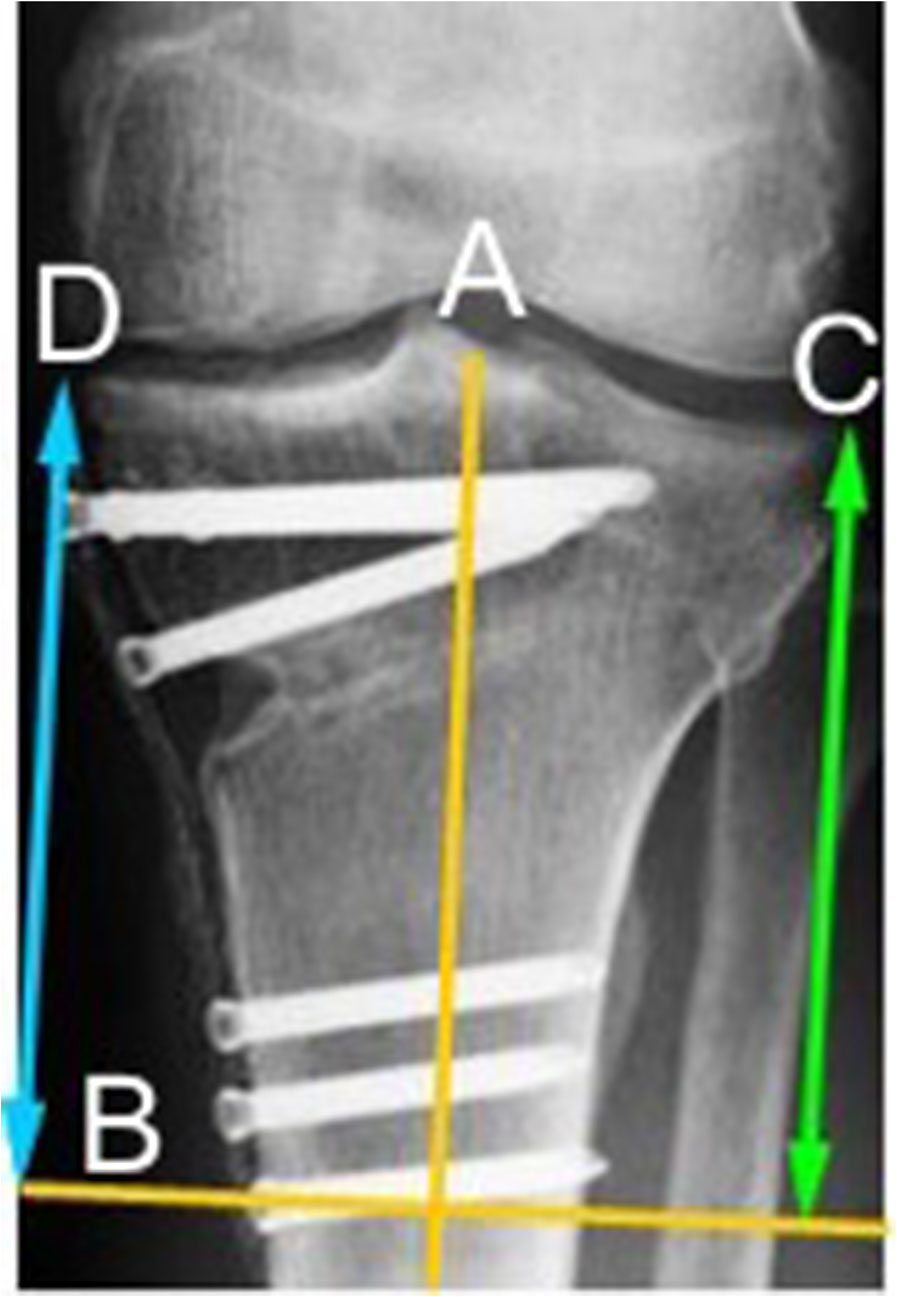
Measurement of loss of correction after owHTO on posterior-anterior radiographs. Line *A*: anatomical axis of the tibia; Line *B*: perpendicular to A through the center of the head of the lowest screw; *C*: lateral joint line edge of the tibia; *D*: medial joint line edge of the tibia. Loss of correction was determined by comparing the distance from point *C*, respectively point *D*, to line *B* in the radiographs after 4 weeks and 12 months

For the analysis of loss of correction, a new method was utilized: A first line (A) was drawn through the anatomical axis of the tibia and a second line (B) through the center of the head of the lowest screw (Fig. 2). Line A and line B form a rectangle. Loss of correction in the front view was determined by comparing the distance from the lateral joint line edge (point C) and the medial joint line edge (point D), respectively, to line B in the radiographs 1 month and 12 months postoperative.

### Statistical analysis

Statistical analysis was performed with JMP, version 14 (SAS Institute Inc., Cary, North Carolina, USA). A Shapiro–Wilk test was done to determine the normal distribution of the samples. The nonparametric Wilcoxon/Kruskal-Wallis test (rank sums) was used to test for differences between more than two groups and the Dunn’s test for post hoc analysis. The Pearson’s chi-squared test was used to analyze differences between the expected frequencies and the observed frequencies for two categorical variables. Linear regression was used to investigate the correlation of two continuous data sets. *P* values of less than 0.05 were considered to indicate statistical significance.

## Results

### Clinical results

This retrospective study was performed using data from patients who underwent owHTO with the 2nd generation PEEKPower HTO plate between 2010 and 2015 and had a minimum follow-up time of 1 year. Three hundred twenty-four patients (346 knees) were included, and 229 men and 95 women with an average age of 55 ± 8.9 years (range 30–76 years). According to WHO classification of the body mass index (BMI), 34 patients (11%) had a normal weight; 150 (46%) patients were overweight and 140 (43%) were obese (Table 1).

**Table 1:** Body mass index (BMI) according to WHO classification of the study patients

Weight	BMI (kg/m^2^)	Number of Patients
Normal weight	19–24.9	34 (10.5%)
Overweight	25–29.9	150 (46.0%)
Obesity I	30–34.9	90 (28.0%)
Obesity II	35–39.9	37 (11.5%)
Obesity III	≥ 40	13 (4.0%)

### Radiographic results

#### Gap size and gap healing time

Bony healing was observed in all patients. The mean gap healing time was 4.0 ± 1.7 months. A delayed bony healing longer than 4 months was observed in 86 knees (24.8%) (Table 2). In 23 patients (6.6%), healing of the osteotomy gap according to the criteria of Apley, Solomon’s, and Grindley took between 6.5 und 14 months. None of the patients underwent revision surgery because of delayed bony healing.

**Table 2:** Time period for bony healing of the osteotomy gap after owHTO

Gap healing time (months)	2–3	3.5–4	4.5–5	5.5–6	6.5–9	9.5–14
Number of knees	169	85	31	38	18	5

**Fig. 3:**
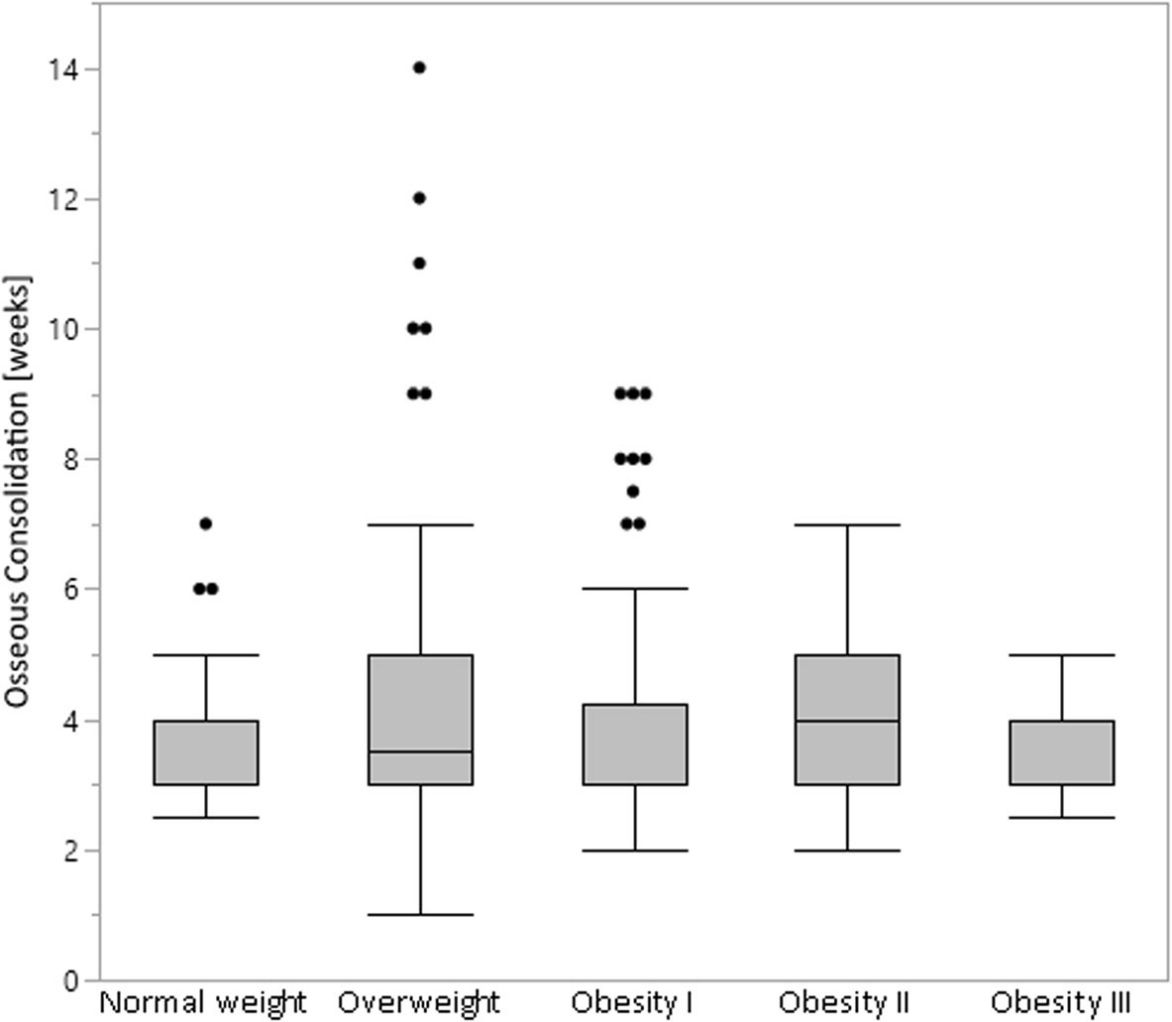
The bony healing time of the osteotomy gap is independent of the patient’s BMI. Normal weight: BMI 19–24.9 kg/m^2^. Overweight: BMI 25–29.9 kg/m^2^. Obesity I: BMI 30–34.9 kg/m^2^. Obesity II: BMI 35–39.9 kg/m^2^. Obesity III ≥ 40 kg/m^2^. The box shows the interquartile range (25–75) with median. Whiskers (error bars) above and below the box indicate the 90th and 10th percentiles. Dots represent outliers

Since recent studies showed inferior outcome of patients with a BMI above 30 for owHTO [28, 29], we analyzed whether overweight resulted in delayed bony healing. However, our study data revealed that the point of osseous consolidation was independent from the patient’s BMI, as no statistically significant increase in the bony healing time could be detected with increasing patient’s BMI (*p* = 0.2302) (Fig  3).

Furthermore, we analyzed if the bony healing depended on the gap size. The average gap size of the study patients was 10 ± 2.7 mm (range 5–17). In 58 cases (16%), the osteotomy gap size was ≥ 12.5 mm. A linear regression showed that the gap size had a significant influence on the time for osseous consolidation (*p* < 0.001). Our data indicate that larger gap sizes result in a prolonged bony healing.

#### Hinge fracture

A lateral hinge fracture is a typical complication of owHTO. According to Takeuchi it is classified into three grades [8]. In our retrospective study, 105 of 346 knees (30%) had a hinge fracture with the majority being classified Takeuchi grade I (Table 3).

**Table 3:** Hinge fractures of study patients classified according to Takeuchi

	Number of knees
No fracture	241 (70%)
Takeuchi I	79 (22.5%)
Takeuchi II	19 (5.5%)
Takeuchi III	7 (2%)

We analyzed the effect of BMI, osteotomy gap size, osteotomy technique (uni-/biplanar), the use of the bone graft substitute Osferion®, and the surgeon on the risk to sustain a hinge fracture. A logistic regression showed that with raising osteotomy gap size, the risk for a hinge fracture was significantly increased in the study population (*p* = 0.0041). At the same time, the BMI (*p* = 0.0850), osteotomy technique (*p* = 0.1370), use of Osferion (*p* = 0.9427) and the surgeon (*p* = 0.2522) were not found to have an effect on the occurrence of a hinge fracture (Pearson’s chi-squared test).

**Fig. 4:**
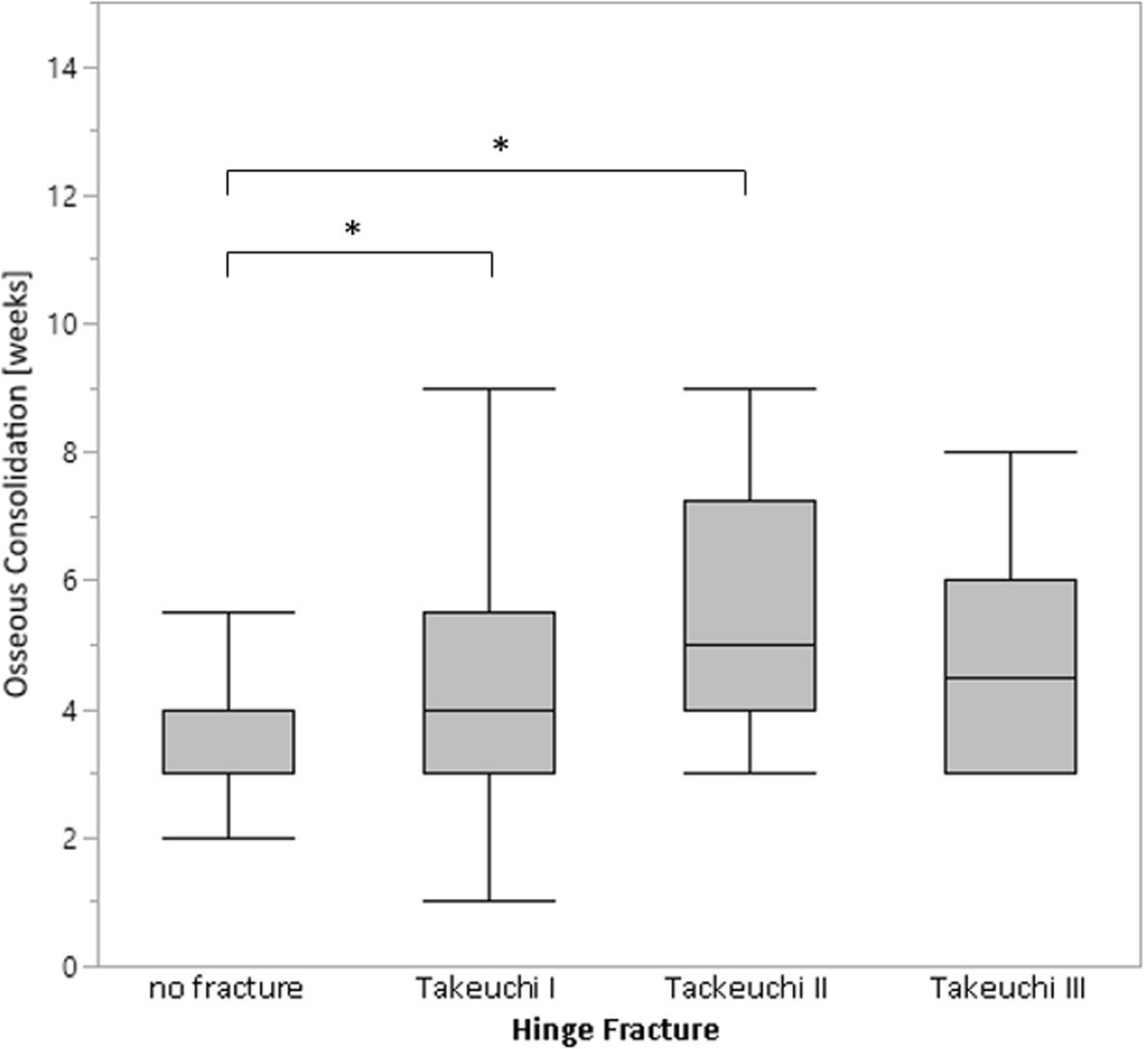
Takeuchi fractures increase the bony healing time after owHTO. Patients with hinge fracture types I and II have a significantly longer healing time than patients without fracture. The box shows the interquartile range (25–75) with median. Whiskers (error bars) above and below the box indicate the 90th and 10th percentiles. *p ≤ 0.05

Furthermore, a significant influence of the hinge fracture on the gap healing time could be determined (*p* ≤ 0.001). A post hoc comparison showed that patients with hinge fractures grades I and II needed a significant longer time for bony healing than patients without a hinge fracture (*p* = 0.0069 and *p* = 0.0002, respectively) (Fig. 4). Interestingly, patients with Takeuchi III fractures had no significantly longer bony healing time in comparison to the other groups (*p* > 0.05).

#### Correction accuracy

We determined the correction accuracy as described in the method section (compare Fig. 2). For 5 knees, the final radiograph was not available and hence, loss of correction could not be analyzed. In 298 knees (87%), there was no loss of correction 12 months after owHTO. Twenty-five knees (7.3%) had a decreased correction height of 1 mm, 10 knees (2.9%) between 1.5 and 2.0 mm, and 2 knees (0.6%) between 2.5 and 3 mm. Interestingly, in 6 knees (1.8%), the correction height at the lateral cortex was increased (1–4 mm) at 12 months postoperative. According the Takeuchi score, these 6 patients had also lateral hinge fractures. It could be shown that the occurrence of a hinge fracture increased the risk for loss of correction accuracy in the total study population (Pearson’s chi-squared test, *p* = 0.0370). The type of lateral hinge fracture (Takeuchi grades I–III), however, had no significant influence (*p* = 0.3410). Likewise, the loss of correction height was independent of the osteotomy gap size (*p* = 0.7247). Overall, 105 knees (30.8%) had a lateral hinge fracture but only 16 of them had a loss of correction height with only 4 (1.2%) of them having a clinically relevant loss of ≥ 3 mm.

#### Complications

**Fig. 5:**
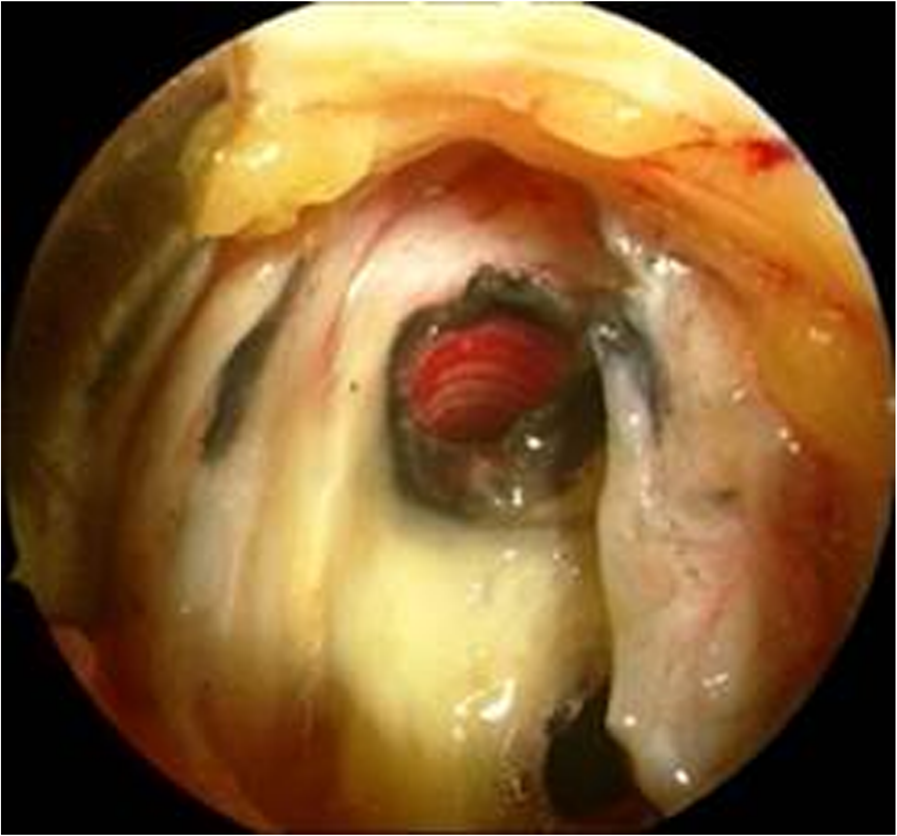
Intraoperative image after removal of the PEEKPower HTO plate with tissue discoloration

During the entire follow-up, none of the patients had an injury of nerve structures with sensory or motor deficit, a screw breakage, or material failure. During the removal surgery, nearly all patients showed a black tissue discoloration around the plate without signs of inflammation or local infection (Fig. 5). Eleven patients (3%) had a wound infection. Four of them were treated with local therapy and antibiotics. Seven further patients (2%) had a deep infection. As a consequence, the implant was removed after 4.5–6 months in these cases. None of the patients with an infection had a loss of correction height.

## Discussion

Several plate fixation systems are available to fix the osseous gap at the proximal tibia, which arises during owHTO. Retrospective studies have shown satisfactory results for many of them with survival rates of 78–90% at 10 years and 56–68% at 15 years after HTO [1, 3, 30–32]. However, frequent complications of owHTO are hardware failures and lateral cortical hinge fractures, which can lead to a delayed union, a non-union, or a loss of correction accuracy [5, 6, 11, 12].

The main finding of this study was that the PEEKPower HTO plate led to an osseous consolidation in all patients with a mean gap healing time of 4.0 ± 1.7 months independent of the presence of hinge fractures or BMI. Despite a hinge fracture rate of 30%, there was no case of non-union and only 4 patients (1.2%) had a loss of correction accuracy with a clinical relevant loss of ≥ 3 mm.

The mean gap healing time of 4 months demonstrated in this study is slightly faster compared to similar devices, as Brosset et al. observed that bone union occurred at 4.5 months on average after owHTO with the TomoFix plate [26]. Reason for the shorter healing time of the PEEKPower HTO plate might be the higher IFM. In our study, 26.5% patients had a delayed osseous healing, which was defined as absence of bony healing on plain radiographs after 4 months according to Miller [10]. Nevertheless, the bone gap healed in this cohort without further surgery 1 year after owHTO in all cases. There was no case of non-union. Differently, Cotic et al. reported for the first generation PEEKPower HTO plate non-union in 12% of the patients (*n* = 26) [20]. In a prospective study (*n* = 28) with the 2nd generation PEEKPower HTO plate, they found only one case of non-union without bone grafting [21]. Noteworthy, in this study, Cotic et al. excluded patients who had a gap size larger than 12 mm because in their opinion additional bone grafting seemed to be necessary using the 2nd generation PEEKPower plate. Several studies suggested that gap filling could protect lateral cortex fracture and loss of correction [33–35]. In our study, patients had an osteotomy gap size ranging between 5 and 17 mm (average 10 ± 2.7 mm), in 58 cases, the gap size was ≥ 12.5 mm. The use of gap filler (Osferion) in 85% of the patients had no significant influence on the bony healing. However, it could be shown that with increasing gap sizes bony healing was significantly prolonged (*p* < 0.001). Overall, our results suggest a clear improvement of the 2nd generation PEEKPower HTO plate compared to the 1st generation regarding the outcomes of bony union. Although it must be noted that studies are difficult to compare because the criteria for delayed union and non-union are not defined uniformly, the 2nd generation PEEKPower plate seems to provide better results when compared to the non-union rate of other owHTO fixation devices. Warden et al. sent questionnaires to all members of the Australian Knee Society asking for the rate of united, delay-united, and non-united owHTOs they performed [11]. Of the reported 182 owHTOs using different fixation plates, 6.6% were classified as delay-united and 1.6% non-united. A large case series after owHTO (*n* = 245) using an internal fixation plate system was performed by Hernigou [31]. There were two patients with delayed unions for whom weight-bearing was delayed for 3 months, and one patient with non-union, which required removal of acrylic bone cement. For the TomoFix HTO plate Brosset et al. reported a non-union rate of 7% [26] and Meidinger et al. of 5.4% [36]. The latter showed that risk factors with a statistically significant influence on the development of a non-union included smoking, body mass index, and fracture of the lateral cortical hinge. In the present study, the majority (89%) of the patients were overweight or obese, but the BMI of the patient had no influence on the positive outcomes. This indicates that owHTO using the PEEKPower plate leads to a bony consolidation also in severely obese patients.

A typical, well-known complication during owHTO is the fracture of the lateral cortex. It increases stress forces and leads to higher demands on fixation systems. Takeuchi et al. presented in 2012 a new classification system for hinge fractures which was used also in this study [8]. However, Takeuchi determined all fractures during surgery or immediately afterwards. In our study, the hinge fracture was identified using the first radiograph taken 4 weeks postoperatively. Previous studies reported hinge fracture rates between 18% and 39% [7–10]. We could show a comparable rate (30%) of lateral hinge fractures compared to the studies mentioned before. Most of our patients had a Takeuchi type I fracture (22%). Similarly to the findings of Takeuchi, we found type II in 5.5% and type III in 2% of the patients [8]. Our study data indicated a significantly increased risk for a hinge fracture with raising osteotomy gap size (*p* = 0.0041). Furthermore, we were able to show that Takeuchi I and II hinge fractures led to increased bony healing times after owHTO with the PEEKPower plate. None of the patients with a hinge fracture exhibited non-union. Mentionable, patients with fracture type III had no significant delayed bone healing time, which may be due to the low number of patients observed with lateral hinge fracture type III. Overall, our results demonstrate that owHTO using the PEEKPower plate leads to satisfactory results even in case of a lateral hinge fracture. Furthermore, we claim that the risk of a lateral hinge fracture is not a typical plate-related result but rather related to the surgical technique.

Many research studies did not focus on the postoperative correction accuracy, but in our opinion, this aspect is one of the most important quality feature for a fixation plate system. Unfortunately, there is no consistent method for measuring the loss of correction height.

Agneskirchner et al. have reported about loss of correction height as a comparison between prescript and achieved correction. However, they did not describe the method of measurement [13, 15, 37]. The present study introduces a new reproducible method to determine the loss of correction in PA radiographs. To our knowledge, this measurement has never been used before. According to this method, 298 patients (87%) had no loss of correction 1 year after owHTO, even those whose implant was removed early after 4.5–6 months because of deep infection. Of the 13% with a loss of correction, only 1.5% had a clinical relevant loss of ≥ 3 mm. We assume that especially the new design of the 2nd PEEKPower HTO plate is responsible for these excellent outcomes. Stoffel compared two different implants (Puddu Plate versus TomoFix Plate) and stated that in case of lateral cortex fracture only the Puddu Plate needed an additional screw fixation in contrast to the rigid long plate fixation system [38]. Agneskirchner concluded the same in his study comparing the biomechanics of four different implants [13]. In particular, he determined that shorter plates provided less stability, leading to lateral cortex fractures and loss of correction. This conclusion is not applicable for this current study.

In our study, overall 7 cases with deep infection (2%) were identified, which is comparable to other studies [8, 9, 26]. Over the entire follow-up period, none of the patients had neither an injury of nerve structures with sensory or motor deficit nor a screw breakage or material failure at the removal of the plate material. During the removal surgery, nearly all patients showed a black tissue discoloration around the plate without signs of inflammation or local infection (Fig. 5). Cotic et al. showed in a previous study that this discoloration has no cytotoxic effect [20].

### Limitations

We are aware that our study has limitations. First, this is a case series without any control group. However, our purpose was in fact to evaluate the radiologic 1-year outcomes of the PEEKPower HTO plate after owHTO in a large patient group. Hence, the chosen study design seems appropriate to collect real-world data. Second, a scientific discussion of the study outcomes was only possible to a limited extent: Other studies analyzing owHTO with a plate fixation system differ in patient population (age, BMI, rehabilitation programs), criteria for delayed or non-union of the bone, and methods for measuring the loss of correction.

## Conclusion

Open wedge HTO using the PEEKPower HTO plate for patients with medial osteoarthritis of the knee in combination with tibial varus deformity leads to excellent bony consolidation also in cases with a hinge fracture, a gap size > 12 mm, and for severely obese patients.

## Data Availability

The datasets used and/or analyzed during the current study are available from the corresponding author on reasonable request.

## Abbreviations

CF-PEEK: Carbon fiber reinforced poly-ether-ether-ketone
IFM: Interfragmentary movement
owHTO: Open wedge high tibial osteotomy
WHOs: World Health Organization

## References

[1] Aglietti P, Buzzi R, Vena LM, Baldini A, Mondaini A (2003). High tibial valgus osteotomy for medial gonarthrosis: a 10- to 21-year study. J Knee Surg.

[2] Jung WH, Chun CW, Lee JH, Ha JH, Kim JH, Jeong JH (2013). Comparative study of medial opening-wedge high tibial osteotomy using 2 different implants. Arthroscopy.

[3] Sprenger TR, Doerzbacher JF (2003). Tibial osteotomy for the treatment of varus gonarthrosis. Survival and failure analysis to twenty-two years. J Bone Joint Surg Am.

[4] Brouwer RW, Bierma-Zeinstra SM, van Raaij TM, Verhaar JA (2006). Osteotomy for medial compartment arthritis of the knee using a closing wedge or an opening wedge controlled by a Puddu plate. A one-year randomised, controlled study. J Bone Joint Surg Br.

[5] Spahn G (2004). Complications in high tibial (medial opening wedge) osteotomy. Arch Orthop Trauma Surg.

[6] van den Bekerom MP, Patt TW, Kleinhout MY, van der Vis HM, Albers GH (2008). Early complications after high tibial osteotomy: a comparison of two techniques. J Knee Surg.

[7] Schröter S, Freude T, Kopp MM, Konstantinidis L, Dobele S, Stockle U (2015). Smoking and unstable hinge fractures cause delayed gap filling irrespective of early weight bearing after open wedge osteotomy. Arthroscopy.

[8] Takeuchi R, Ishikawa H, Kumagai K, Yamaguchi Y, Chiba N, Akamatsu Y (2012). Fractures around the lateral cortical hinge after a medial opening-wedge high tibial osteotomy: a new classification of lateral hinge fracture. Arthroscopy.

[9] Martin R, Birmingham TB, Willits K, Litchfield R, Lebel ME, Giffin JR (2014). Adverse event rates and classifications in medial opening wedge high tibial osteotomy. Am J Sports Med.

[10] Nelissen EM, van Langelaan EJ, Nelissen RG (2010). Stability of medial opening wedge high tibial osteotomy: a failure analysis. Int Orthop.

[11] Warden SJ, Morris HG, Crossley KM, Brukner PD, Bennell KL (2005). Delayed- and non-union following opening wedge high tibial osteotomy: surgeons’ results from 182 completed cases. Knee Surg Sports Traumatol Arthrosc.

[12] Nawas HT, Vansadia DV, Heltsley JR, Suri M, Montgomery S, Jones DG (2016). Factors affecting the union of opening wedge high tibial osteotomy using a titanium wedge plate. Ochsner J.

[13] Agneskirchner JD, Freiling D, Hurschler C, Lobenhoffer P (2006). Primary stability of four different implants for opening wedge high tibial osteotomy. Knee Surg Sports Traumatol Arthrosc.

[14] Spahn G, Muckley T, Kahl E, Hofmann GO (2006). Biomechanical investigation of different internal fixations in medial opening-wedge high tibial osteotomy. Clin Biomech (Bristol, Avon).

[15] Lobenhoffer P, Agneskirchner J, Zoch W (2004). Open valgus alignment osteotomy of the proximal tibia with fixation by medial plate fixator. Orthopade.

[16] Staubli AE, De Simoni C, Babst R, Lobenhoffer P (2003). TomoFix: a new LCP-concept for open wedge osteotomy of the medial proximal tibia–early results in 92 cases. Injury.

[17] Doornink J, Fitzpatrick DC, Madey SM, Bottlang M (2011). Far cortical locking enables flexible fixation with periarticular locking plates. J Orthop Trauma.

[18] Roderer G, Gebhard F, Duerselen L, Ignatius A, Claes L (2014). Delayed bone healing following high tibial osteotomy related to increased implant stiffness in locked plating. Injury.

[19] PEEKPower High tibial osteotomy plate – white paper LA2-0118-EN_A [Internet]. 2013 [cited 2019 Feb 07]. Available from https://d1psc3qesfsa61.cloudfront.net/pdfs/cnCqQ7xBN02lzwE8SjFidA/cnCqQ7xBN02lzwE8SjFidA.pdf?Expires=1549526558&Signature=aFK8z4e1wCh6uHjlECMRB2FZU5eUGQelq31cSedi8UvsijIFr-E-bJ923KOMg8Dtcbfz4vhyX8FBLnIxipCjGm8fRkX-597SRko5U065hZY1Vwj5Rhtqlmcc5wc48EPIdd-fkv7YML2R81XVCLTOXp6gw-xTKp5jgEDj6NHIhnBXqNAFI5UgL8El~DySDaijEHvooanBZYeWQarqHTePUAJbGzi3LnjJwVWhlXNi7hzTGzn5I7zt412L2gbnL6HN90hf~dwCjMjCB1kQkN1UiZgorb-qkLn1Zfy~Rkwb5Je5fUkJPHlWzjIs2lnhuJbwEVPl2BoA0FdBN~hQ7jKWkA__&Key-Pair-Id=APKAJMGJRW6JX5OBM5LA.

[20] Cotic M, Vogt S, Hinterwimmer S, Feucht MJ, Slotta-Huspenina J, Schuster T (2015). A matched-pair comparison of two different locking plates for valgus-producing medial open-wedge high tibial osteotomy: peek-carbon composite plate versus titanium plate. Knee Surg Sports Traumatol Arthrosc.

[21] Cotic M, Vogt S, Feucht MJ, Saier T, Minzlaff P, Hinterwimmer S (2015). Prospective evaluation of a new plate fixator for valgus-producing medial open-wedge high tibial osteotomy. Knee Surg Sports Traumatol Arthrosc.

[22] Lobenhoffer P, Agneskirchner JD (2003). Improvements in surgical technique of valgus high tibial osteotomy. Knee Surg Sports Traumatol Arthrosc.

[23] Lobenhoffer P, De Simoni C, Staubli AE (2002). Open-wedge high tibial osteotomy with rigid plate fixation. Tech Knee Surg.

[24] Franco V, Cipolla M, Gerullo G, Gianni E, Puddu G (2004). Open wedge osteotomy of the distal femur in the valgus knee. Orthopade.

[25] Miniaci A, Ballmer FT, Ballmer PM, Jakob RP (1989). Proximal tibial osteotomy. A new fixation device. Clin Orthop Relat Res.

[26] Brosset T, Pasquier G, Migaud H, Gougeon F (2011). Opening wedge high tibial osteotomy performed without filling the defect but with locking plate fixation (TomoFix) and early weight-bearing: prospective evaluation of bone union, precision and maintenance of correction in 51 cases. Orthop Traumatol Surg Res OTSR.

[27] Solomon L, Warwick DJ, Nayagam S. Apley and Solomon’s concise system of orthopaedics and trauma. 4th ed: CRC Press; 2014.

[28] Floerkemeier S, Staubli AE, Schroeter S, Goldhahn S, Lobenhoffer P (2014). Does obesity and nicotine abuse influence the outcome and complication rate after open-wedge high tibial osteotomy? A retrospective evaluation of five hundred and thirty three patients. Int Orthop.

[29] Yokoyama M, Nakamura Y, Onishi T, Hirano K, Doi M (2016). Healing period after open high tibial osteotomy and related factors: can we really say that it is long?. SpringerPlus.

[30] Choi HR, Hasegawa Y, Kondo S, Shimizu T, Ida K, Iwata H (2001). High tibial osteotomy for varus gonarthrosis: a 10- to 24-year follow-up study. J Orthop Sci.

[31] Hernigou P, Ma W (2001). Open wedge tibial osteotomy with acrylic bone cement as bone substitute. Knee.

[32] Hui C, Salmon LJ, Kok A, Williams HA, Hockers N, van der Tempel WM (2011). Long-term survival of high tibial osteotomy for medial compartment osteoarthritis of the knee. Am J Sports Med.

[33] Gaasbeek RD, Toonen HG, van Heerwaarden RJ, Buma P (2005). Mechanism of bone incorporation of beta-TCP bone substitute in open wedge tibial osteotomy in patients. Biomaterials.

[34] van Hemert WL, Willems K, Anderson PG, van Heerwaarden RJ, Wymenga AB (2004). Tricalcium phosphate granules or rigid wedge preforms in open wedge high tibial osteotomy: a radiological study with a new evaluation system. Knee.

[35] Kumar G, Dunlop C (2011). Case report: a technique to remove a jammed locking screw from a locking plate. Clin Orthop Relat Res.

[36] Meidinger G, Imhoff AB, Paul J, Kirchhoff C, Sauerschnig M, Hinterwimmer S (2011). May smokers and overweight patients be treated with a medial open-wedge HTO? Risk factors for non-union. Knee Surg Sports Traumatol Arthrosc.

[37] Maffulli N, Loppini M, Longo UG, Denaro V, Oliva F (2013). Bovine xenograft locking Puddu plate versus tricalcium phosphate spacer non-locking Puddu plate in opening-wedge high tibial osteotomy: a prospective double-cohort study. Int Orthop.

[38] Stoffel K, Stachowiak G, Kuster M (2004). Open wedge high tibial osteotomy: biomechanical investigation of the modified Arthrex Osteotomy Plate (Puddu Plate) and the TomoFix Plate. Clin Biomech (Bristol, Avon).

